# My Motor-Cycling Experiences: By a Country Practitioner

**Published:** 1912-05-11

**Authors:** 


### THE PRACTITIONER'S RELAXATIONS AND HOBBIES.

### My Motor-Cycling Experiences: By a Country Practitioner.

The strong boom in motor cycling brought about by that noteworthy practical advance, perfected magneto ignition, is now being largely taken advantage of by medical men. The practitioner of not too mature years, whose district is not wholly urban, finds certain mutual advantages to himself and patients in the new form of high-speed locomotion, at any rate for most months in the year. As regards the doctor's side of the matter, he can travel four or five times the same distance for the same expenditure in petrol, while tyres and repairs come very much cheaper; also he can leave his man at home to do gardening or odd jobs. As regards the patients' interest, the uncanny quickness with which a telegram will fetch the doctor with a good motor-cycle is certainly reassuring to apprehensive souls. Appearances must be sacrificed a little, but happily in the twentieth century everybody is getting beyond the "top-hat nonsense," and is coming to see that in the matter of dress, suitability to the business in hand is much more important than compliance with conventional ideas of the becoming and the impressive. People of all social conditions must be getting used to seeing the doctor speed up to their door in dusty overalls (easily doffed); at least it is to be hoped so, for in the country the number of practitioners who pretty well lay up their car during the summer is rapidly growing.

It is, however, a pity that the makers of these machines—some at least—do not give sufficient elementary instructions with them. I have known, indeed, of two extreme instances, where men bought motor-cycles and mounted them without it ever having been impressed upon them that these things want oil regularly and frequently. They rode gaily on until the unfortunate cylinders seized. One of them brought an action, because he said the vendors had never told him to oil it. The result I never heard; he must somehow have missed getting with his purchase the usual accompanying handbook which deals thoroughly with so vital a point as lubrication. But what I here refer to is the use of paraffin while the engine is still hot, whenever a stop of longer than a quarter of an hour is made. Some makers mention this in passing, but for all beginners, and in the colder months for all riders whatsoever, it is surely essential. Until they began the routine of injecting paraffin liberally into the compression tap and working the engine a little as each machine came in, they often had complaints from angry and perhaps not too expert riders who could not make their hirelings start at all. My own rather painful early experience with a very popular two-speed twin lightweight bears this out completely. Cure of a gummed-up piston was found to be a much more troublesome business than the process of prevention above described. Moreover, if one had forgotten to fill the paraffin can, cure became well nigh impossible, for petrol is for this particular purpose nothing like so good as its humbler relative. In that embarrassing event it became necessary to wheel to a steepish hill, run down with free engine, and then suddenly "shift into the high." This was successful in starting firing, but in this way I broke a timing pinion, and (I think from this) another time got a cylinder seized far from all help; and, being thus left with only one wing to fly with, had to push about ten miles out of the twenty home.

This brings up the evergreen discussion of the relative advantages of twin lightweights and of 3½ h.p. single cylinders. In an adventure such as that just described the former would score, for the lightest of 3½'s would have had to be left at the nearest cottage. In mud, too, the former are steadier. But after all, all motor-cycles, when there is no luggage on the carrier, are infinitely less liable to skid than the uninitiated, remembering their pedal cycles, imagine. One should waste no respect on the nerves of motor-cyclists, barring scorchers and dashing "cornermen." However, as against twins, there is two of everything to go wrong, and when riding in rain (a thing doctors have to think of) the more exposed sparking plug soon short-circuits. There is a shield sold to prevent this, but it is another lodgment for mud, and another complication to look after. The lighter tyres fitted to the lightweights wear out sooner, and of course these machines are slower, although, in my experience at least, less liable to belt-slipping on hills. This last is a great bugbear, and a medical neighbour of mine is getting rid of a well-known make simply for this reason, the strength of the engine seeming to go rather to overcoming the friction of the belt-pulley than to speed. Another evil the beginner will probably soon encounter is overheating. But the cure is simple; would that all stoppages were as easily remedied! Sit quiet in the saddle for five minutes and then go on again rather slower, giving oil rather more frequently than before. If the tendency to overheat becomes pronounced, it probably means that the carbon deposit on the head of the piston wants chipping or burning off.

But perhaps one has dwelt too much upon the drawbacks of the pastime. For it is something of a pastime the sense of possession of power in such small bulk being quite exhilarating. As another neighbour (who worked his practice first with a hunter and a wonderful old pony, then with a car, but latterly with a solo machine and—he is a good married man—an 8 h.p. Chater-Lea for sidecar purposes) says, driving a motor-cycle is much more interesting and "sporting" than driving a car. For crowding two days' work of a scattered practice into one, so as to get a day free, without using a lot of petrol and taking it out of one's car, there is nothing like a fast motor-cycle. Where it fails a little as yet is that it suits the summer season better than the winter, and so is less of a stand-by just when the work of a practice is at its busiest.

